# A lecturer’s voice quality and its effect on memory, listening effort, and perception in a VR environment

**DOI:** 10.1038/s41598-024-63097-6

**Published:** 2024-05-30

**Authors:** Isabel S. Schiller, Carolin Breuer, Lukas Aspöck, Jonathan Ehret, Andrea Bönsch, Torsten W. Kuhlen, Janina Fels, Sabine J. Schlittmeier

**Affiliations:** 1 Work and Engineering Psychology, RWTH Aachen University, Aachen, 52066 Germany; 2 Institute for Hearing Technology and Acoustics, RWTH Aachen University, Aachen, 52074 Germany; 3 Visual Computing Institute, RWTH Aachen University, Aachen, 52074 Germany

**Keywords:** Audio-visual language processing, Virtual reality, Voice quality, Human behaviour, Psychology

## Abstract

Many lecturers develop voice problems, such as hoarseness. Nevertheless, research on how voice quality influences listeners’ perception, comprehension, and retention of spoken language is limited to a small number of audio-only experiments. We aimed to address this gap by using audio-visual virtual reality (VR) to investigate the impact of a lecturer’s hoarseness on university students’ heard text recall, listening effort, and listening impression. Fifty participants were immersed in a virtual seminar room, where they engaged in a Dual-Task Paradigm. They listened to narratives presented by a virtual female professor, who spoke in either a typical or hoarse voice. Simultaneously, participants performed a secondary task. Results revealed significantly prolonged secondary-task response times with the hoarse voice compared to the typical voice, indicating increased listening effort. Subjectively, participants rated the hoarse voice as more annoying, effortful to listen to, and impeding for their cognitive performance. No effect of voice quality was found on heard text recall, suggesting that, while hoarseness may compromise certain aspects of spoken language processing, this might not necessarily result in reduced information retention. In summary, our findings underscore the importance of promoting vocal health among lecturers, which may contribute to enhanced listening conditions in learning spaces.

## Introduction

Our ability to process speech and retain informational content is crucial in many everyday scenarios. In educational settings, for example, effective listening is one of the cornerstones for successful learning. However, schools and universities rarely provide optimal listening conditions. Some of the challenges faced by the students are background noise^1,2^ (including peer conversations^3^), inadequate room acoustics^4–6^, and poor speaker voice quality^7^. In the presence of such adversities, visual cues provided by the speaker (e.g., lip movements) can help listeners complement masked auditory information^8–10^. Given the multimodal facet of spoken language processing, research on acoustic obstacles to effective listening should not be confined to audio-only experiments. Here, we employed audio-visual virtual reality (VR) to study the effect of a lecturer’s voice quality on university students’ retention of heard information, their listening effort, and their overall listening impression.

University professors primarily communicate with their students through spoken language. As professional voice users, their occupation involves a high vocal load, which puts them at an elevated risk of developing dysphonia^11^. Dysphonia, also referred to as hoarseness, is often characterized by a rough, breathy, and strained voice quality^12^. Like background noise, dysphonia impairs the quality of the speech signal, but during production, not transmission. According to a recent meta-analysis^7^, approximately 41% of university professors develop voice disorders, including dysphonia, compared to merely 6% among the general population^13^. The consequences of a lecturer’s dysphonia are experienced not only by the individuals themselves but also by their students. Several studies have demonstrated that people with dysphonia are perceived more negatively in terms of personal characteristics than healthy talkers^14,15^. Furthermore, the speech of individuals with dysphonia is less intelligible, particularly in noisy environments^16^.

Acoustically, an impaired voice can be regarded as phonation noise, originating from the vocal source (the larynx) and distorting the speech signal in an unpleasant manner^17^. That is, irregular or incomplete vocal fold oscillations result in a devoicing of otherwise voiced consonants^18^ and in consonant contrasts becoming less distinct^16^. Vowel intelligibility, particularly for low vowels like /ɑ/(as in ‘bath’) and /ɔ/ (as in ‘thought’), can also be diminished in dysphonic individuals, possibly due to reduced acoustic energy above 1 kHz^19^. Such speech signal degradations may result in increased listening effort for dysphonic voices. Listening effort can be referred to as “the attention and cognitive resources required to understand speech” (McGarrigle et al.^20^, p. 2). An explanation for the elevation of listening effort under (acoustically) challenging conditions is offered by Rönnberg et al.’s^21^ multimodal Ease of Language Understanding (ELU) model. The ELU model posits that in the presence of adverse acoustic conditions, listeners must allocate more cognitive resources to actually perceive and comprehend what they hear. If the available resources are exceeded, listeners make more errors and need more time to process information.

Voice issues among lecturers are widely recognized, yet our understanding of dysphonic speech effects on students’ perception and cognition is incomplete. This gap is partly due to the predominance of audio-only experiments, primarily conducted with children in primary school settings. Most of of these studies indicate that when the speaker’s voice is dysphonic, pupils perform significantly weaker in phoneme discrimination tasks^22,23^, speech-intelligibility tasks^24^, and listening comprehension tasks^25,26^ compared to when listening to a healthy voice. These effects may vary depending on task difficulty^27^ and working memory capacity^28^. While the impacts of dysphonic voices on younger children are well-documented, our understanding of how these findings apply to adult university students is limited to two studies^14,29^. Applying a Dual-Task Paradigm, Imhof et al.^14^ observed that exposure to creaky voice, also known as vocal fry, increased university students’ listening effort and decreased their ability to retain informational content from narrated stories. In contrast, Schiller et al.^29^ reported no significant effect of hoarse voice quality on university students’ performance in a similar task. The underlying reasons for these deviant findings remain speculative and could relate to specific voice characteristics and other methodological aspects. Importantly, these two studies as well as the aforementioned studies solely presented the speech material through auditory means. This approach overlooks the potential impact of visual cues, which are integral to everyday communication settings. We are not aware of any study that has applied an audio-visual setting to assess the effect of a speaker’s voice quality on university students’ auditory cognition. A tool that could provide valuable insights into the importance of visual cues in such scenarios is immersive virtual reality (VR).

Employing audio-visual VR as an alternative to traditional audio-only listening experiments allows for the investigation of participants’ auditory cognition in a highly controlled and at the same time more plausible setting^9,30^. Compared to the isolated presentation of speech via the auditory modality, audio-visual VR offers a more precise representation of how individuals might encounter similar listening conditions in real life and react to them. Moreover, VR has already established itself as a valuable tool for research into speech production^31–34^. For instance, Remacle et al. demonstrated that a virtual classroom can elicit similar speech behaviors among teachers as a real classroom^32^, and can provide a training environment for teachers to practice specific speech techniques^33^. In summary, VR is a versatile method for conducting audio-visual studies on speech communication, particularly valuable in education, where teaching and learning contexts are typically multimodal.

The aim of this VR study was to investigate the effect of a virtual professor’s voice quality on students’ ability to recall content from narratives, their listening effort, and their overall listening impression. For this purpose, participants—drawn from a sample of university students—were immersed in a virtual seminar room environment with typical ambient noise. They faced a female professor standing at a lectern in the front of the room and reading out narratives using either a typical voice quality or a hoarse voice quality. Three hypotheses were tested:

- **H1 (Voice quality and memory):** Participants will remember less content information from audio-visually presented narratives when the virtual professor’s voice quality is hoarse compared to typical.
- **H2 (Voice quality and behavioral listening effort):** When performing a secondary task while listening to the narratives, participants will show more erroneous and/or slower responses in that task as they listen to the hoarse voice compared to the typical voice, signaling a higher listening effort.
- **H3 (Voice quality and listening impression):** Participants will rate the overall listening impression more negatively when they evaluate the condition in which they listened to the hoarse voice compared to the healthy voice.

To test these H1 and H2, we conducted a Dual-Task Paradigm using Heard-Text Recall (HTR^35,36^) as a primary task, and a vibrotactile secondary task^37^. Overall listening impression with regard to the two voice qualities (H3) was assessed with a questionnaire. A detailed description of the HTR, the vibrotactile task, and the questionnaire is provided in the “Methods” section.

**Figure 1:**
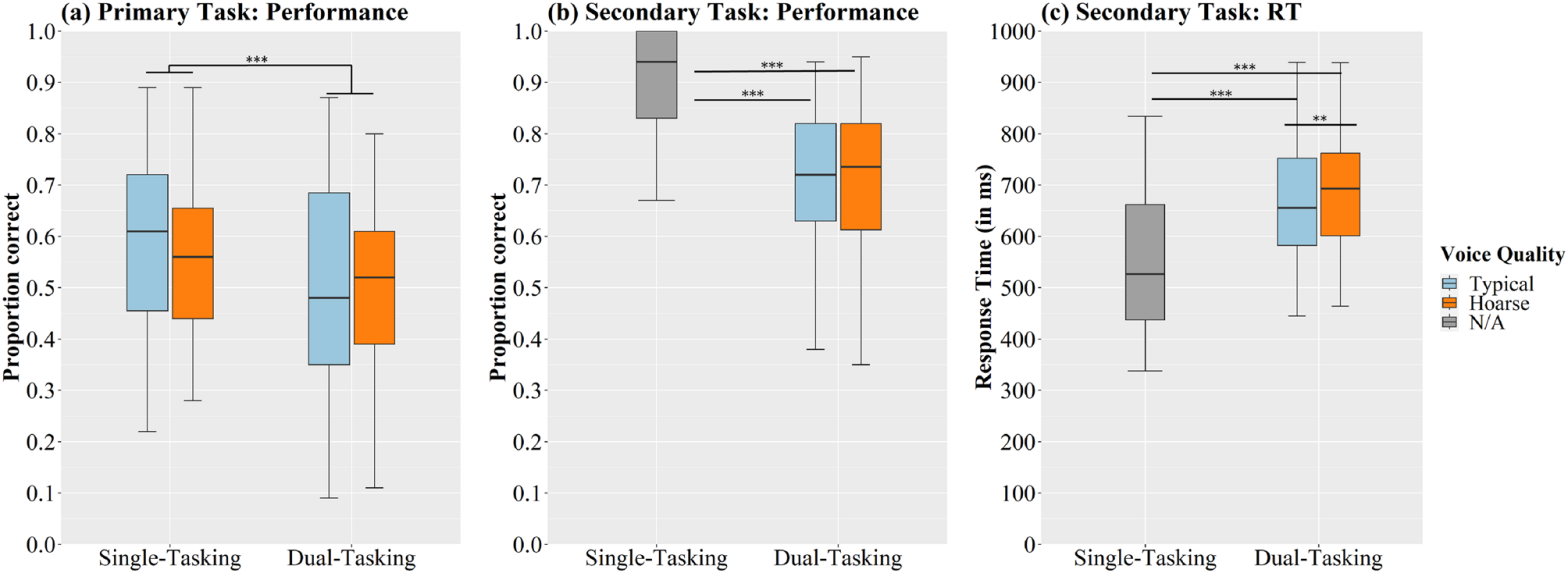
Boxplots illustrating the distribution of (**a**) primary task performance, (**b**) secondary task performance, and (**c**) response times in the secondary task for both single- and dual-tasking conditions, and as a function of voice quality. The color grey for N/A indicates that no speech was presented in this condition. ***p < 0.001, **p < 0.01.

**Figure 2:**
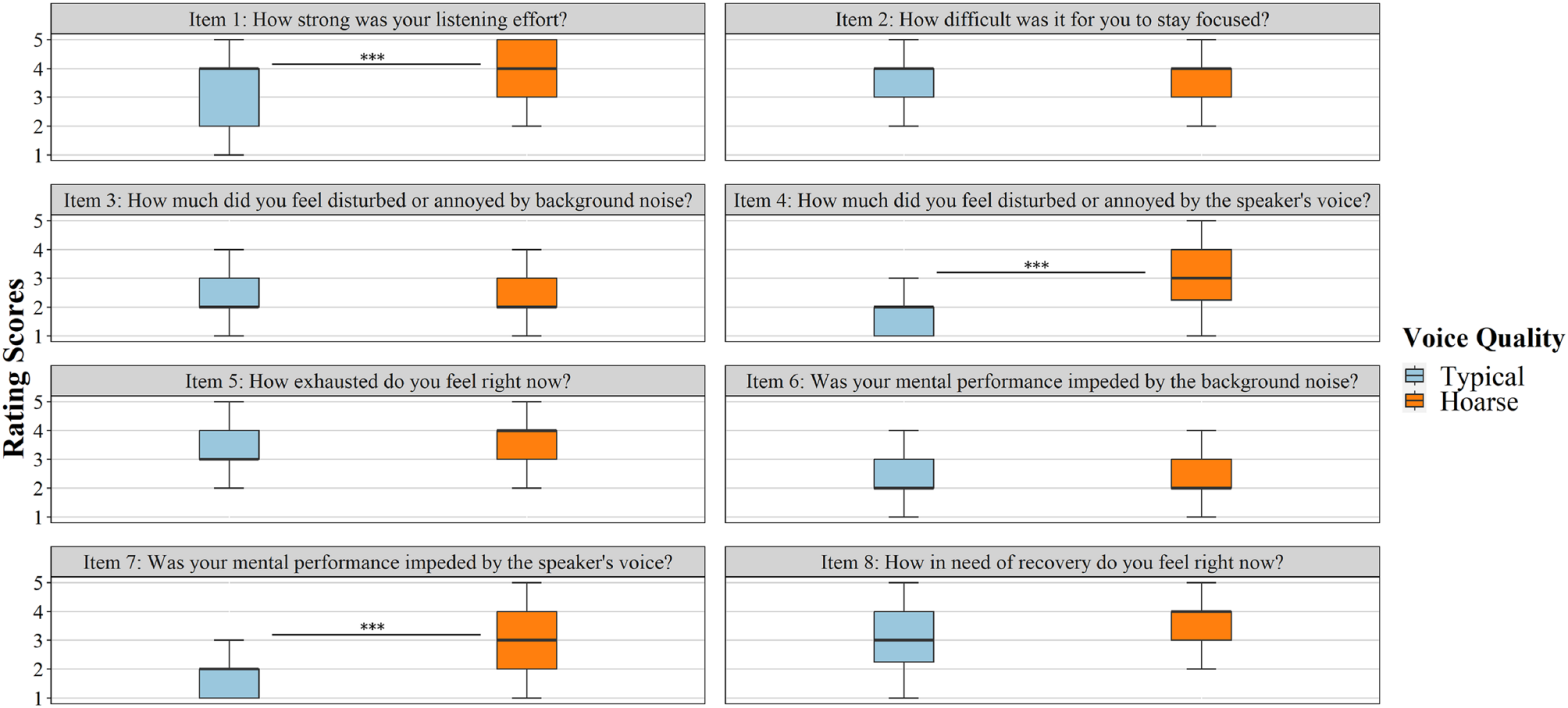
Listening impression ratings for each voice quality and each questionnaire item. Rating scores range from 1 (*not at all*) to 5 (*extremely*). ***p < 0.001.

**Figure 3:**
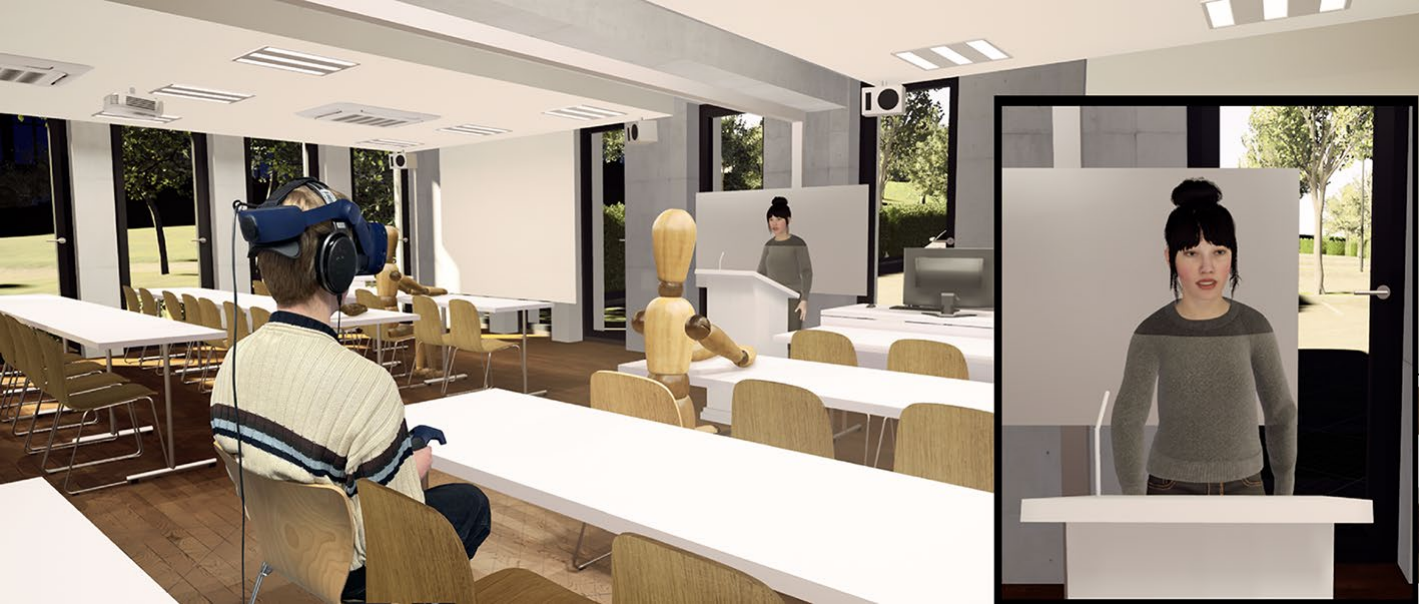
Participant immersed in the virtual seminar room with the virtual professor up front. The close-up of the professor is depicted here for illustrative purposes—it was not displayed on the HMD.

**Figure 4:**
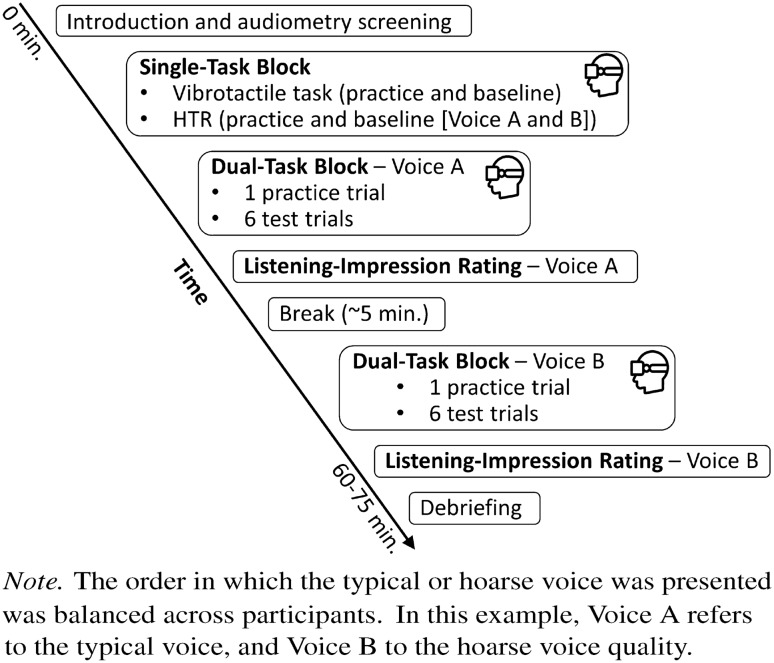
Flow chart of the experimental procedure.

## Results

### Memory for content information

Participants’ memory for content information from stories was measured based on their performance in the HTR. Figure 1a shows HTR performance as a function of *voice quality* and *task mode*. In terms of *voice quality*, participants yielded a slightly higher proportion of correct answers when listening to the typical voice ($M = 0.54, \ SD = 0.19$) compared to the hoarse voice ($M = 0.51, \ SD = 0.17$). However, according to the results of our GLMM, the effect of *voice quality* on HTR performance was not significant ($\chi ^2(1) = 2.69, p = .1$), with the complete model output presented in Table 1. Instead, *task mode* (single- vs. dual-tasking) significantly affected participants’ task performance ($\chi ^2(1) = 43.31, p < 0.001$). A post-hoc test of this effect revealed that participants performed significantly better during the single-task condition as opposed to when they were concurrently engaged in the secondary task ($\textit{z}\text {-ratio} = 6.58, \textit{p} < 0.001$). The mean proportions of correct answers were 0.56 (*SD* = 0.18) for single-tasking and 0.50 (*SD* = 0.18) for dual-tasking. As also shown in Table 1, there was a significant impact of *trial* (referring to each subsequent text presentation) on HTR performance. This effect demonstrates that, independently of *voice quality*, participants’ became better at recalling the heard information across trials ($\chi ^2(1) = 13.41, p < 0.001$).

### Behavioral listening effort

Behavioral listening effort was measured in the form of participants’ performance (proportion of correct responses) and response times (RT) in the secondary (vibrotactile) task. Results of these two outcome measures are shown in Fig. 1b (performance) and Fig. 1c (RT), highlighting that, during dual-tasking, the speaker’s voice did not affect secondary task performance but had an impact on response time. The GLMM output is presented in Table 2 (performance) and Table 3 (RT). RT in the vibrotactile task was significantly affected by *task condition* (single-tasking, dual-tasking with typical voice, and dual-tasking with hoarse voice; $\chi ^2(2) = 197, p < 0.001$). Post-hoc analysis revealed significantly faster responses during single-tasking compared to both dual-tasking conditions (all *p*’s < 0.001). Most importantly, when dual-tasking, participants responded significantly faster to the vibrations as they were exposed to the typical compared to the hoarse voice ($\textit{z}\text {-ratio} = -3.52, \textit{p} = 0.001$). Regarding performance, the effect of *task condition* was also significant ($\chi ^2(2) = 105, p < 0.001$), but not with regard to the speaker’s voice quality. Post-hoc analysis demonstrated that participants were significantly better during single-tasking compared to both dual-tasking conditions (all *p*’s < 0.001). However, whether they were exposed to the typical or hoarse voice during dual-tasking did not make a difference ($\textit{z}\text {-ratio} = -1.69, \textit{p} = 0.21$).

### Listening impression

According to the results of the listening-impression questionnaire, participants judged their listening impression as more negative for the hoarse voice compared to the typical voice. Figure 2 depicts boxplots of the rating scores for each questionnaire item as a function of *voice quality*. Wilcoxon signed-rank tests revealed that participants rated the hoarse-voice condition significantly lower than the typical-voice condition for three items (Item 1, Item 4, and Item 7). Specifically, they indicated perceiving a stronger listening effort (Item 1: $V = 43, \ p < 0.001, \ d = 0.55$ [medium effect size]), greater annoyance (Item 4: $V = 0,\ p < 0.001, \ d = 1.11$ [large effect size]), and a stronger impediment to their cognitive performance (Item 7: $V = 21,\ p < 0.001,\ d = 1.02$ [large effect size]) in relation to the hoarse voice. Rating scores for the remaining items did not significantly vary with *voice quality*. Specifically, listening to the hoarse voice did not significantly affect participants’ perceived ability to stay focused (Item 2), noise annoyance (Item 3), level of exhaustion (Item 5), cognitive-performance impediment in relation to noise (Item 6), and need for recovery (Item 8), with *p*’s ranging between 0.13 and 0.36.

**Table 1:** GLMM results for performance in the primary task as predicted by voice quality, task mode, and trial.

Fixed effects	Estimate	SE	z	95% CI	p
Intercept	0.29	0.16	1.76	− 0.04, 0.62	0.079
Voice quality					
Typical	Reference				
Hoarse	− 0.09	0.05	− 1.64	− 0.20, 0.02	0.10
Task mode					
ST	Reference				
DT	− 0.48	0.07	− 6.66	− 0.62, − 0.33	$<0.001$***
Trial	0.03	0.01	3.66	0.02, 0.05	$<0.001$***

Random effects	Variance	SD			
Participant (Intercept)	0.67	0.82			
Item (Intercept)	0.06	0.25			
Question: Item	0.86	0.92			
Nr. of observations: 7200, Participant = 50; Item: 16; Question: 144					

*ST* single-tasking, *DT* dual-tasking; confidence intervals calculated with the Wald method; Model equation: performance $\sim$ voice quality + task mode + trial + (1|participant) + (1|item/question), family = binomial, link function = logit

**Table 2:** GLMM results for performance in the secondary task as predicted by task condition.

Fixed effects	Estimate	SE	z	95% CI	p
Intercept	2.38	1.18	12.94	1.94, 2.73	$<0.001$***
Task condition					
ST	Reference				
DT typical voice	− 1.43	0.12	− 12.16	− 1.67, − 1.21	$<0.001$***
DT hoarse voice	− 1.36	0.12	− 11.54	− 1.59, -1.13	$<0.001$***

Random effects	Variance	SD			
Participant (intercept)	0.55	0.74			
Vibration pattern (intercept)	0.04	0.20			
Nr. of observations: 11,457, Participant = 50; Vibration pattern: 4					

*ST* single-tasking, *DT* dual-tasking; confidence intervals calculated with the Wald method; Model equation: performance $\sim$ task condition + (1|participant) + (1|vibration_pattern), family = binomial, link function = logit

**Table 3:** GLMM results for response time in the secondary task as predicted by task condition.

Fixed effects	Estimate	SE	z	95% CI	p
Intercept	6.28	0.08	82.36	6.13, 6.43	$<0.001$***
Task condition					
ST	Reference				
DT typical voice	0.26	0.02	15.64	0.23, 0.29	$<0.001$***
DT hoarse voice	0.30	0.02	17.94	0.27, 0.33	$<0.001$***

Random effects	Variance	SD			
Participant (intercept)	0.03	0.18			
Vibration pattern (intercept)	0.02	0.14			
Nr. of observations: 8287, Participant = 50; Vibration pattern: 4					

*ST* single-tasking, *DT* dual-tasking; confidence intervals calculated with the Wald method; Model equation: RT $\sim$ task condition + (1|participant) + (1|vibration_pattern), family = Gamma, link function = log

**Table 4:** Perceptual and acoustic evaluation of the typical and hoarse voice quality.

	Typical voice	Hoarse voice
	Median (IQR)	Median (IQR)
Perceptual voice-quality analysis (GRBAS^a^ rating)		
G (grade)	0 (0)	3 (1)
R (roughness)	0 (0)	1 (1)
B (breathiness)	0 (1)	3 (1)
A (asthenia)	0 (1)	2 (1)
S (strain)	1 (1)	3 (0)
I (instability)	0 (0)	2 (1)
Acoustic voice-quality analysis (AVQI^b^)		
AVQI score	1.92	4.86

^a^The GRBAS(I) scale^63,64^ is a well-established instrument for perceptual voice-quality assessment. Each GRBAS(I) parameter is rated on a 4-point scale, ranging from 0 (normal) to 3 (severe disturbance). The G parameter represents the overall grade of dysphonia and is composed of the remaining parameters. ^b^The Acoustic Voice Quality Index (AVQI^65^) is a metric to objectively evaluate voice quality, based on various acoustic parameters. AVQI scores range from 0 to 10, with lower values indicating a better, and higher values indicating a poorer voice quality. The German cut-off between a typical and dysphonic voice is 3.05^66^

## Discussion

The prevalence of voice disorders in university professors is 41%^7^. Similar to background noise, the consequence of a speaker’s dysphonia is an acoustically degraded speech signal^16,18^. This poses a challenge for listeners, particularly for students attending seminars or lectures where focused listening and learning are tightly linked. Studies on the impact of dysphonia on spoken language processing remain scarce and have been restricted to presenting just the speech signal in audio-only experiments without any visual speech-related cues^14,29^. However, situations where we both hear and see the speaker, including their lip movements, are very common. In the present study, we have carried out an audio-visual VR experiment assessing the effect of a lecturer’s voice quality on university students’ memory for heard content, listening effort, and overall listening experience. We found that, while memory for heard content did not vary with the speaker’s voice quality, response times in a secondary task performed concurrently with the listening task were significantly longer when participants listened to the hoarse voice compared to the typical voice. This indicates an increased listening effort under the hoarse-voice condition. Moreover, participants’ overall listening impression was rated more negatively when the speaker’s voice quality was hoarse.

Our hypothesis (H1) that participants would recall less content information from audio-visually presented narratives when the professor’s voice quality was hoarse compared to typical was not confirmed. Although there was a slight decrease in HTR performance under the hoarse-voice condition, it was not statistically significant. This contrasts with Imhof et al.’s^14^ audio-only study, which indicated that listening to an impaired voice negatively impacts both comprehension and memory. It also diverges from established theories such as the ELU model^21^, according to which acoustic degradations should diminish cognitive resources that are then not available for retaining information. Interestingly, our findings replicate Schiller et al.’s^29^ results from an audio-only study, where no significant effect of voice quality on recall was observed. In both Schiller et al.^29^ and the present study, mean recall performance was descriptively lower for the hoarse voice condition (56%^29^ and 51% respectively) compared to the typical voice condition (58%^29^ and 54% respectively), yet this was not statistically significant. Perhaps our null finding is related to the population from which participants were drawn—university students with assumed strong working memory, as working memory is predictive of academic achievement^38^. Working memory capacity plays an important role in an individual’s ability to process acoustically degraded speech^21^ and our participants might have been particularly adept at compensating for dysphonic speech. Populations with less developed working memory capacity, such as pupils, may encounter greater difficulty retaining information from dysphonic speech^39^, especially those with weaker executive functions^26^. However, the discrepancy with Imhof et al.’s study remains unexplained, despite similar participant demographics, warranting further research.

When engaged in a secondary task concurrently to the listening task, participants required significantly longer RT in the hoarse-voice compared to the typical-voice condition. This finding is in support of our second hypothesis (H2) stating that secondary-task performance would be weaker and/or slower under the hoarse-voice exposure. It also aligns with the well-accepted theory that cognitive resources are limited and deplete more quickly under degraded listening conditions^21^. We posit that upon hoarse-voice exposure, participants allocated more cognitive resources to maintain the same performance level in the primary task, consequently becoming slower in the secondary task. In Schiller et al.^29^, neither performance nor RT in the secondary task was affected by the speaker’s voice quality. Some methodological variances may account for this difference in findings. Schiller et al.^29^ used a visual number-judgment task as a secondary task and observed that participants performed near-to-perfect (i.e. ceiling effect) in all three conditions (single-task baseline, dual-task with typical voice, and dual-task with hoarse voice). Perhaps with only the auditory modality engaged in the primary task, the overall cognitive load was lower, and, thus, number judgments were not sufficiently challenging as a secondary task. This could have prevented the detection of meaningful differences regarding the speaker’s voice quality. In the present study, the secondary task engaged the sensory modality alongside the visual and auditory modalities that were engaged for carrying out the primary task. The fact that no ceiling effects were found suggests that vibrotactile tasks might be more effective for measuring behavioral listening effort than number judgment—at least during audio-visual speech presentation. Another methodological difference relates to voice quality. In Schiller et al.^29^, participants listened to a different female speaker with different vocal characteristics. That speaker’s imitated hoarse voice was moderately impaired, characterized by a high degree of roughness, strain, and instability. In the present study, the speaker’s imitated hoarse voice was slightly more impaired (moderately to severely), characterized by a high degree of breathiness and strain. These vocal characteristics might have made it more effortful to listen to. Thoroughly investigating this hypothesis would require an extensive acoustic analysis, which was, however, beyond the scope of this study.

Finally, our participants evaluated their listening impression more negatively regarding the speaker’s hoarse voice compared to the typical voice, which aligns with our hypothesis H3. The analysis of the subjective rating data indicates that the hoarse voice induced a significantly greater listening effort, more annoyance, and a significant perceived impediment to mental performance. This corroborates the conclusions drawn from our behavioral data (RTs in the secondary task), which also pointed to heightened listening effort with the hoarse voice. It also mirrors the results of our audio-only study^29^, wherein the same items received more negative ratings for the hoarse voice. While hoarseness compromised some aspects of subjective speech perception, not all items were impacted by voice quality. Participants’ ability to stay focused, their level of exhaustion, and their need for recovery appeared unaffected by the speaker’s voice. Moreover, noise annoyance and perceived noise-induced performance decrements were comparable in both voice conditions. As this also aligns with our previous findings^29^, we carefully conclude that a speaker’s voice affects students’ subjective listening impression similarly in both audio-only and audio-visual conditions.

Reflecting on our findings, several limitations and perspectives for future research should be discussed. We acknowledge that, despite our efforts to replicate realistic listening conditions by using audio-visual VR, the controlled environment in which we collected our data remains artificial. Participants were not explicitly instructed to engage with the virtual seminar room as if it were real, nor did we use a fiction contract^40^ for outlining rules or expectations regarding the VR setting. A fiction contract is particularly useful and can improve the sense of immersion when participants are expected to interact with the virtual scene or agents in certain ways^31^. In our study, participants were only required to listen to, but not interact with, the virtual professor, which is why we opted for a more general instruction and task description. Another limitation relates to the simplicity of the animation of the virtual professor and the audio-visual dynamics within the seminar room, which primarily focused on our main research question—the impact of voice quality. Due to the reduced visual cues, participants might have relied more heavily on auditory information when processing the virtual professor’s speech signal. However, this enhanced reliance on the auditory input likely mirrors scenarios commonly experienced in seminar rooms and lecture halls, where the lecturer’s visibility may be constrained, especially for students sitting in the back rows. Expanding on our research, future studies might involve more accurate animations of gaze, gesture, and breathing (see, e.g., Ehret et al.^41^) to study their impact on, for example, speech perception, attitude towards the lecturer, or audio-visual learning. Moreover, as outlined in the methods, an oversight during the study implementation created an audio delay of about 140 ms. This delay went unnoticed during data collection, and none of the participants remarked on any audio-visual discrepancies. Notably, various studies indicate that even audio delays up to about 180–250 ms still allow for an effective integration of audio-visual speech stimuli^42–46^. Nevertheless, there is a possibility that this slight stimulus onset asynchrony increased participants’ cognitive load and perceived listening effort. Moving forward, we will refine our synchronization and implementation methods to ensure even higher accuracy in audio-visual presentation. Finally, our findings do not allow us to conclude how dysphonia in male speakers might affect listeners. We chose a female speaker for our study because dysphonia is more prevalent in females than males^7,13^.

To conclude, our findings indicate that a lecturer’s hoarse voice may increase listening effort and be perceived more negatively than a typical (healthy) voice. This has implications for the educational context and beyond. In settings where complex information is communicated through speech, such as in lessons, seminars, and lectures, the speaker’s vocal health may be crucial. Although a lecturer’s poor voice quality may not directly hamper students’ performance, it may slow down information processing. A speaker’s hoarseness can also make listening—and probably learning—less pleasant and subjectively less effective. Recognizing the integral role that vocal health plays in the transmission of knowledge, it becomes clear that improving listening conditions in learning spaces entails more than enhancing room acoustics and reducing noise; it also necessitates promoting lecturers’ vocal health. This involves educating these occupational groups about vocal hygiene and efficient voice use^47–49^. Additionally, it requires the early identification of individuals with vocal concerns to prevent further deterioration, as recommended in a recent meta-analysis^7^.

## Methods

An audio-visual VR experiment was conducted in a sound-proof booth at the Institute of Psychology at RWTH Aachen University. Participants were tested individually, immersed into the scene of a virtual seminar room utilizing a head-mounted display (HMD, HTC Vive Pro Eye; see Fig. 3) and Sennheiser HD 650 headphones. We applied a within-subject design to assess the effect of the virtual professor’s *voice quality* (typical vs. hoarse) on students’ memory, listening effort, and listening impression. Approval for the study, which was conducted in accordance with all relevant regulations, was granted by the ethics committee of the Faculty of Arts and Humanities (ref. 2022_016_FB7_RWTH Aachen). Informed consent was obtained from all participants before the experiment commenced.

### Participants

Fifty-seven university students participated in the study. However, our data analysis was carried out on a final set of 50 participants (33 females, 17 males), aged between 18 and 43 years (*M* = 25, *SD* = 5). All participants were proficient German speakers with either native fluency or equivalent language skills (self-report), reported normal or corrected-to-normal vision, and normal hearing ($\le$ 20 dB HL between 500 Hz and 4 kHz according to a pure-tone audiometry screening, performed with an ear 3.0 audiometer [Auritec]). Data from the remaining seven participants were excluded because of technical issues (*n* = 4), participants’ decision to drop out (*n* = 2), and poor German skills (*n* = 1).

### Tasks

In a virtual seminar room (Fig. 3), participants performed a Dual-Task Paradigm, consisting of a primary (listening) task and a secondary task in the haptic domain. Participants performed both tasks alone (single-tasking) and concurrently (dual-tasking). The primary task, Heard Text Recall (HTR^35^), assessed their memory for content information. It involved listening to a virtual female professor reading out short stories (about 60 s each) about different family members, including details on their relationships with one another, professions, and leisure activities. The professor’s voice quality varied across two blocks; in one experimental block, it was typical, and in the other it was hoarse. An audio-visual demonstration of the HTR in both voice qualities is provided in Supplementary Video V1, with English subtitles added post-hoc. After each story, participants had to answer nine content-related questions in 1–2 words each. While the questions were presented in written form in the virtual environment, participants provided their answers verbally. No time limit was set for the responses. Participants’ answers were coded as 1 (*correct*) and 0 (*incorrect*) by the experimenter.

The secondary task was a vibrotactile task^37^, designed to measure behavioral listening effort. Participants were equipped with two HTC Vive controllers, one in each hand, through which they repeatedly received four different vibration patterns (short-short, long-long, short-long, long-short). After each vibration pattern, their task was to indicate whether the two vibrations were identical in length (long-long, short-short) or different (short-long, long-short) by pressing the trigger button of the right or left HTC Vive controllers. If participants did not respond within 2 s, the program automatically logged a missing response. Responses in the secondary task were automatically coded as 1 (*correct*) and 0 (*missed/incorrect*) by the system. Listening effort was quantified based on performance and RTs from correct trials (i.e., time from stimulus offset to button press).

Participants also completed a pen-paper listening-impression questionnaire for both the typical and the hoarse voice quality, which required removing the HMD after each voice-quality block. Eight items (questions) had to be rated on a 5-point scale, ranging from 1 (*not at all*) to 5 (*extremely*): (1) How strong was your listening effort?, (2) How difficult was it for you to stay focused?, (3) How much did you feel disturbed or annoyed by background noise?, (4) How much did you feel disturbed or annoyed by the speaker’s voice?, (5) How exhausted do you feel right now?, (6) Was your cognitive performance impeded by the background noise?, (7) Was your cognitive performance impeded by the speaker’s voice?, and (8) How in need of recovery do you feel right now?. The same questionnaire was previously used by Schiller et al.^29^.

### Voice recordings and audio-visual rendering

The HTR texts used in this study were recorded in a typical and a hoarse voice quality. Recordings took place in a hemi-anechoic chamber ($length \times width \times height = 11 \times 5.97 \times 4.5 {\text { m}}^3$) at RWTH Aachen University’s Institute of Hearing Technology and Acoustics to allow for acoustically dry recordings. The speech was recorded using a DPA 4066 CORE omnidirectional microphone. Apart from the audio recordings, we utilized an iPhone XR and the Apple ARKit to synchronously record the speaker’s articulatory movements (see, e.g., Ehret et al.^50^), limited to facial expressions and lip movements, to subsequently animate the corresponding ECA’s face (Embodied Conversational Agent^51^). To facilitate the later synchronization of the audio and video signals, the speaker produced visible and audible claps. The speaker was the first author (I.S.), a 34-year-old female voice researcher with a background in speech-language pathology. She initially read the stories in her typical voice and then imitated a dysphonic voice. Both voice qualities underwent a perceptual and acoustic voice-quality evaluation, the results of which are presented in Table 4. In summary, the typical voice was confirmed to be healthy, while the imitated hoarse voice exhibited a moderate to severe degree of dysphonia, particularly characterized by perceived breathiness and strain. Each recorded HTR text was loudness adjusted based on EBU R 128 standards^52^.

For the experiment, the virtual professor’s speech—the HTR texts in both voice qualities—was binaurally rendered and adjusted to a presentation level of 65 dBA, using the room acoustic simulation software RAVEN and the Virtual Acoustics auralization framework^53^. While immersed in the scene, participants were also exposed to realistic ambient sounds, played back at a signal-to-noise ratio of approximately 13 dBA. This SNR can be understood as representing a mild to moderate noise disturbance. The ambient sounds consisted of ventilation noise, incomprehensible speech, and other disruptive sounds like paper rustling, chair movement, and keyboard typing. Binaural recordings of these sounds were made with an artificial head^54^, placed in an occupied seminar room at the Institute of Hearing Technology and Acoustics at RWTH Aachen University. The same ambient sounds were presented in Schiller et al.^29^. Since the participants answered orally and Sennheiser HD 650 open headphones were used, they were able to hear their own voices during the experiment. To account for the reverberation in the audio-visually presented seminar room, the participants’ voices were convolved with the room impulse response of the seminar room, and this reverberant speech was played back on the participants’ headphones to enhance the impression of being in the audio-visual VR scene.

Regarding the visual VR scene, we used a virtual replica of the same seminar room^55^ in which we had recorded the ambient sounds. This allowed us to visually map the sounds to the respective sources. For example, to provide a visual representation of the irrelevant sounds coming from peer students, we used six wooden mannequins representing these students in VR. They were placed in the same seating positions as the real students occupying the seminar room during the noise recordings. We embedded only abstract, static representations for these peers, to avoid adverse effects from them not moving naturally in sync with the sounds, as this synchronous animation was beyond the scope of this project. The virtual professor, hence, the ECA reading out the HTR texts in the two voice qualities, was represented by a female MetaHuman (www.unrealengine.com/metahuman). Her body posture and gestures were identical for both voice qualities. The virtual professor was strategically placed within the room, standing at a lectern directly within the view of the participants (Fig. 3). Her gaze was animated following an approach by Pejsa et al.^56^ to shift between her notes on the lectern and the audience, including the participant seated in the third row, featuring a read-out style. Her lip movements represented direct mappings from the motion-capturing recordings of the real speaker. It should be noted that an oversight in implementation resulted in a minor asynchrony between the visual animation and the speech signal, specifically an audio delay of approximately 140 ms. According to the speech perception literature, however, minor audio delays up to about 180–250 ms still permit audio-visual integration^42–46^. Therefore, participants most probably perceived the lip movements and speech as largely congruent. The study was implemented using the *Unreal Engine 4.27* and study control was realized using the *StudyFramework*^57^.

### Procedure

The entire VR experiment took approximately 60–75 min, including an actual immersion time of approximately 45 min. The experimental procedure is depicted in a flow chart (Fig. 4). After an introduction and the audiometry screening, participants were equipped with the head-mounted display (HMD) and headphones, and were seated in the sound-proof booth at a table matching the virtual one (see Fig. 3). Upon an initial eye calibration, they began the experiment with the single-task block. It started with a practice phase of the vibrotactile task, followed by a baseline phase in which participants’ only task was to indicate whether the two vibrations presented in the respective trial were identical in length or different. Preceded by an HTR practice text, we then measured HTR baseline performance for both voice qualities. This measurement was based on two texts for each voice quality. Next, in the first of two dual-task blocks, participants performed both the vibrotactile task and the HTR in parallel. Six HTR texts with the corresponding questions were presented, preceded by a practice text. Texts were balanced across both dual-task blocks, and the presentation of voice quality in each dual-task block was balanced among participants. Specifically, half of the participants completed the first block in the typical-voice condition and the second block in the hoarse-voice condition, while the other half followed the reverse sequence. Upon finishing the first dual-task block, participants removed the HMD and completed the listening impression questionnaire for the respective voice quality. After a short break, the experiment continued with the second dual-task block, featuring a new set of HTR texts, presented in the other voice quality. Again, HTR and vibrotactile tasks were presented in parallel. After completing this block, participants removed the HMD and filled out the listening impression questionnaire again. The experiment ended with a debriefing.

### Statistical analysis

Data analysis was carried out with R^58^, mainly by fitting a generalized linear mixed-effects model (GLMM) in a forward model-selection procedure. GLMMs were specified using the *lme4* package^59^. Whenever post-hoc analyses were applicable, they were conducted using the emmeans package^60^ and applied the Tukey’s HSD test to adjust the significance level to account for multiple comparisons. Subjective data from the listening-impression questionnaire were analyzed with Wilcoxon signed-rank tests. Cohen’s *d* was used to estimate effect sizes of differences between means.

To assess the impact of *voice quality* on recall (H1), we modeled a GLMM with a binomial distribution and logit link function. As fixed factors, we considered *voice quality* (typical vs. hoarse), *task mode* (single-tasking vs. dual-tasking), *trial* (referring to each subsequent text presentation), and their interactions. As random factors, we considered *item* (referring to each individual text), *question* (referring to each question), and a random intercept for *participant* to account for the repeated-measures design.

To test the effect of *voice quality* on behavioral listening effort (H2), as measured based on secondary task performance and RTs, we specified separate GLMMs for each outcome. For the GLMM modeling performance, we considered the fixed factors *task condition* (referring to single-tasking, dual-tasking under the typical-voice condition, and dual-tasking under the hoarse-voice condition) and *trial*. As random factors, we considered *vibration pattern* (referring to the four different patterns), *trial*, and a random intercept for each *participant*. Regarding the outcome response time, we first cleaned the data before specifying the GLMM. Specifically, we considered RTs below 150 ms invalid and excluded them, following Whelan et al.’s^61^ suggestion. Moreover, we removed RTs outside the range of 2 SDs from the mean, as proposed in Berger et al.^62^. This resulted in the removal of 492 RT values, representing 5.9% of the data from correct trials. The fixed and random factors considered for the GLMM modeling RT were the same as those for the GLMM modeling performance. Finally, to assess the effect of *voice quality* on listening impression (H3), we used Wilcoxon signed-rank tests. These data were not normally distributed, precluding an analysis with parametric tests. We calculated separate Wilcoxon tests for each item, comparing the rating scores for the typical voice quality to the rating scores for the hoarse voice quality.

### Supplementary Information

Supplementary Video 1.

Supplementary Information.

## Data Availability

The datasets generated and analyzed during the current study are available from the corresponding author on reasonable request.
