# Supplementary material for: A lecturer’s voice quality and its effect on memory, listening effort, and perception in a VR environment
Source: Sci Rep. 2024 May 30;14:12407. doi: 10.1038/s41598-024-63097-6 (PMC11137055; doi:10.1038/s41598-024-63097-6)
Supplement: Supplementary file 2 — Supplementary Information. [file 41598_2024_63097_MOESM2_ESM.pdf]

Supplementary video V1: Audio-visual demonstration of the HTR

Note. This is a supplementary video for the article " A Lecturer's Voice Quality and Its Effect on Memory, Listening Effort, and Perception in a VR Environment" It provides an audio-visual demonstration of the HTR in both typical and hoarse voice conditions, with English subtitles added post-hoc. The speech material is spoken by the first author (I.S.), a 34-year-old female voice researcher with a background in speech-language pathology.
